# A Pilot Randomized Controlled Trial to Explore Cognitive and Emotional Effects of Probiotics in Fibromyalgia

**DOI:** 10.1038/s41598-018-29388-5

**Published:** 2018-07-19

**Authors:** Pablo Roman, Angeles F. Estévez, Alonso Miras, Nuria Sánchez-Labraca, Fernando Cañadas, Ana B. Vivas, Diana Cardona

**Affiliations:** 1 Departamento de Enfermería, Fisioterapia y Medicina, Universidad de Almería, Almería, Spain; 2 Departamento de Enfermería, Universitat Jaume I, Castellón, Spain; 3 CERNEP Research Center, Universidad de Almería, Almería, Spain; 4 Departamento de Psicología, Universidad de Almería, Almería, Spain; 5 Independent Researcher, Almería, Spain; 6 Psychology Department, The University of Sheffield International Faculty, City College, Sheffield, Greece

## Abstract

It has recently been found that microbes in the gut may regulate brain processes through the gut microbiota–brain axis, which modulates affection, motivation and higher cognitive functions. According to this finding, the use of probiotics may be a potential treatment to improve physical, psychological and cognitive status in clinical populations with altered microbiota balance such as those with fibromyalgia (FMS). Thus, the aim of the present pilot study with a double-blind, placebo-controlled, randomised design was to test whether a multispecies probiotic may improve cognition, emotional symptoms and functional state in a sample of patients diagnosed with FMS. Pain, impact of FMS, quality of life, anxiety and depressive symptoms were measured during the pre- and post-intervention phases; participants also completed two computerised cognitive tasks to assess impulsive choice and decision-making. Finally, urinary cortisol concentration was determined. To our knowledge, this is the first study that explore the effect of a multispecies probiotic in FMS patients. Our results indicated that probiotics improved impulsivity and decision-making in these patients. However, more research is needed to further explore the potential effects of probiotics on other cognitive functions affected in FMS as well as in other clinical populations.

## Introduction

Recent studies have shown that intestinal homeostasis may directly affect brain functioning, and consequently modulate affection, motivation and higher cognitive functions^1–4^. It has been proposed that microbes in the gut regulate brain processes through a bidirectional communication network known as the gut microbiota—brain axis (GBA)^5–7^. Communication in this axis occurs via three different pathways: neural (mainly by the vagus nerve and the enteric nervous system), endocrine (cortisol) and immune (cytokines)^5–7^. Several experimental approaches have been used to study the modulatory effects of gut microbiota on gut–brain interactions, including gut microbial manipulation with antibiotics, faecal microbial transplantation and germ-free animal models (revised in Mayer *et al*.^8^). Overall, the results from these preclinical experiments suggest that the microbiota and its metabolites are likely involved in modulating behaviours and brain processes via the GBA, such as stress responsiveness, anxiety, depression and pain modulation^8^.

This emerging line of research has the potential to open new avenues in the treatment of disorders related to brain dysfunction^3^. One such new development is the use of probiotics. A probiotic preparation is a microorganism that beneficially affects the health of the host organism when ingested in an adequate dose^9^. Probiotic consumption appears to exert a myriad of beneficial effects, including enhancement of immune response, balancing of colonic microbiota or reduction of faecal enzymes implicated in cancer initiation, among others^10^. In a recent review, the term psychobiotics was coined to describe the beneficial effects of probiotics in patients suffering from psychiatric illnesses^11^. However, this is a very new field, and there is currently little evidence in support of the hypothesis that cognitive and emotional processes can be influenced by probiotics via the GBA^12,13^. In one of the few double-blind placebo-controlled studies testing the effect of probiotics on mood, results showed that a daily intake of a milk yogurt containing *Lactobacillus casei* Shirota during three weeks improved the mood of participants who had initially worse levels of mood^14^. Two other studies have also reported positive effects of probiotics on anxiety and mood in healthy and moderately stressed human volunteers relative to a placebo-control group^15,16^. Interestingly, a recent study has shown that probiotics can improve depression, increase quality of life and reduce limbic activity to negative emotional stimuli in patients with irritable bowel syndrome^17^.

Recent accumulating evidence has suggested that probiotics, a non-invasive treatment with not known harmful side effects, could improve the physical, psychological and cognitive state of clinical populations with altered microbiota balance. One clinical population that fits into this category are patients suffering from FMS^18^. Nowadays, FMS is defined as a chronic disorder characterised by widespread musculoskeletal pain accompanied by symptoms such as morning stiffness, moderate or severe fatigue, depression, headaches and sleeping disorders^19–21^. Emotional and mood problems are also common in FMS^22–25^. Another prevalent complaint is reduced mental performance^26–31^, being attention, episodic memory and working memory the cognitive domains that appear to be most frequently affected in this population^29,32^. These deficits are mainly observed in complex tasks^26^, particularly those involving distraction or competition with other sources of information which tap on inhibitory mechanisms (e.g., Correa *et al*.^33^ and Leavitt and Katz^34^). FMS patients have also shown poor performance in tasks assessing abstraction, cognitive flexibility^35^ and decision-making^35,36^.

The aim of the present study was to test whether a multispecies probiotic could improve cognition, emotional symptoms and functional state in a population diagnosed with FMS. Regarding to cognition, we decided to focus on two impulsivity constructs (impulsive choice and decision-making) since previous research has suggested a link between impulsivity and FMS^33,35^. The two-choice task and the Iowa gambling task has been widely used in the literature to measure the construct of impulsive choice and emotion-based decision-making, respectively. Although this is a relatively new field of research and there are few studies investigating the effect of probiotics on human cognition, based on the evidence discussed above, we hypothesised that FMS patients would show better performance on these two tasks after the treatment with probiotics. We also expected to observe a beneficial effect of the intervention on the emotional (e.g., depression and anxiety) and physiological measures (free cortisol concentration in urine).

## Results

### Demographic, questionnaires and physiological measures

Table 1 shows the participant characteristics for each group (probiotic versus placebo). No significant group differences were observed for gender distribution [χ^2^ (1, N = 31) = 0.44, p = 0.51], age [t(29) = 1.62, p = 0.12], time with FMS diagnosis [t(29) = 0.05, p = 0.96], years of formal education [t(29) = 0.30, p = 0.76], and BMI [t(29) = −0.34, p = 0.73].

**Table 1:** Demographic characteristics in the Probiotic and Placebo groups.

Demographic characteristics		
	Probiotic	Placebo
Participants (Female:Male)	16 (15:1)	15 (13:2)
Age (years)	55.00 (2.09)	50.27 (2.03)
Time with Fibromyalgia (years)	8.56 (1.47)	8.47 (1.50)
Formal education (years)	12.75 (0.95)	12.27 (1.29)
BMI (kg/m^2^)	29.40 (1.64)	30.23 (1.63)

Mean scores and standard error of the means (in parentheses) are shown.

Table 2 shows a summary of pre- and post-intervention scores on VAS, FIQ, SF-36, BDI, STAI, and MMSE and the mean measures of cortisol free urine for the probiotic and placebo groups.

**Table 2:** Mean scores and standard error of the means (shown in parentheses) on the questionnaires and physiological measures in the Probiotic and Placebo groups. p values indicate the Time by Group interaction.

Questionnaires and Physiological outcomes					
	Pre-intervention		Post-intervention		*p*-value^a^
	Probiotic	Placebo	Probiotic	Placebo	
VAS	6.69 (0.41)	7.50 (0.50)	5.49 (0.38)	6.05 (0.62)	0.72
FIQ	60.92 (2.90)	68.18 (4.63)	55.06 (4.61)	56.15 (5.20)	0.17
SF-36					
General Health	27.81 (3.19)	24.00 (4.09)	34.84 (4.00)	26.67 (5.49)	0.31
Emotional Role	39.58 (12.25)	13.33 (7.13)	43.75 (11.27)	37.78 (12.54)	0.14
Physical Function	39.27 (6.49)	32.33 (5.56)	50.63 (6.77)	42.33 (6.43)	0.78
Physical Role	3.13 (2.13)	5.00 (5.00)	18.75 (8.39)	23.33 (8.61)	0.81
Corporal Pain	23.44 (3.64)	18.33 (4.72)	32.97 (5.94)	30.67 (5.22)	0.75
Vitality	24.06 (3.98)	13.33 (4.04)	31.88 (4.74)	25.67 (6.05)	0.43
Social Function	36.72 (5.87)	32.50 (6.43)	46.88 (6.51)	47.50 (6.92)	0.51
Health Evolution	42.75 (3.61)	31.47 (5.15)	52.00 (4.68)	44.80 (6.02)	0.44
BDI	21.75 (2.10)	30.73 (3.33)	18.88 (2.20)	24.93 (4.07)	0.33
STAI					
State	34.32 (1.53)	33.07 (0.94)	36.07 (3.25)	37.33 (3.79)	0.60
Trait	40.19 (2.87)	44.07 (3.05)	37.19 (2.91)	40.67 (3.59)	0.90
MMSE	28.50 (0.42)	28.47 (0.42)	28.44 (0.42)	28.73 (0.28)	0.50
Cortisol	2531.65 (88.51)	2664.86 (99.37)	1611.76 (70.73)	1802.15 (63.54)	0.70

^a^p-values for Time by Group interaction from repeated measures ANOVAs.

The total score of the pain evaluation (VAS scores) did not reveal any significant effects (ps > 0.05). Also, the ANOVAs showed that the main effect of Group and Time by Group interaction were not significant for any of the measures (FIQ, SF-36, BDI, STAI at both state and trait scales, and MMSE) (ps > 0.05) (see Table 2). Only a main effect of Time was observed for the total score of the FIQ [F(1, 29) = 10.05, p = 0.036, ηp^2^ = 0.257], BDI [F(1, 29) = 8.61, p = 0.006, ηp^2^ = 0.229] and the SF-36, both for the General Health total score [F(1, 29) = 5.20, p = 0.03, ηp^2^ = 0.152] and for the emotional role dimension total score [F(1, 29) = 4.51, p = 0.04, ηp^2^ = 0.135].

Finally, the results from the ANOVA performed on the cortisol free urine levels revealed a main effect of Time [F(1, 28) = 75.26, p = 0.000, ηp^2^ = 0.729]. However, the main effect of Group and the Time by Group interaction did not reach statistical significance (ps > 0.05).

### Cognitive tasks

**Figure 1:**
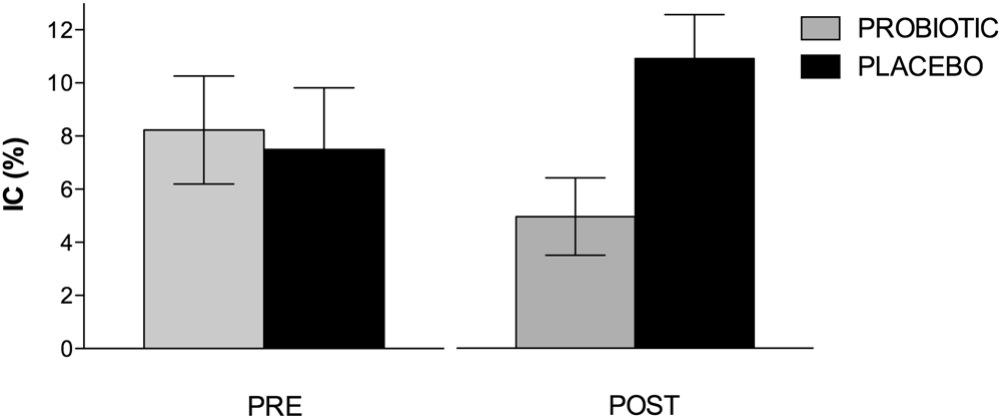
Mean impulsive choices (IC; short-delay) on the two-choice task. Data are presented as the mean and standard error (error bars).

On the two-choice task (a measure of impulsive choice), the number of impulsive choices made by both groups was analysed. The repeated measures ANOVA showed a significant Group by Time interaction [F(1, 21) = 5.53, p = 0.029, ηp^2^ = 0.208]. The analysis of the interaction revealed that both groups showed a similar number of impulsive choices during the pre-intervention assessment (see Fig. 1). However, the probiotic group presented a significantly reduced number of impulsive choices after the treatment [F(1, 21) = 7.24, p = 0.014, ηp^2^ = 0.256]. No other significant effect was observed.

**Figure 2:**
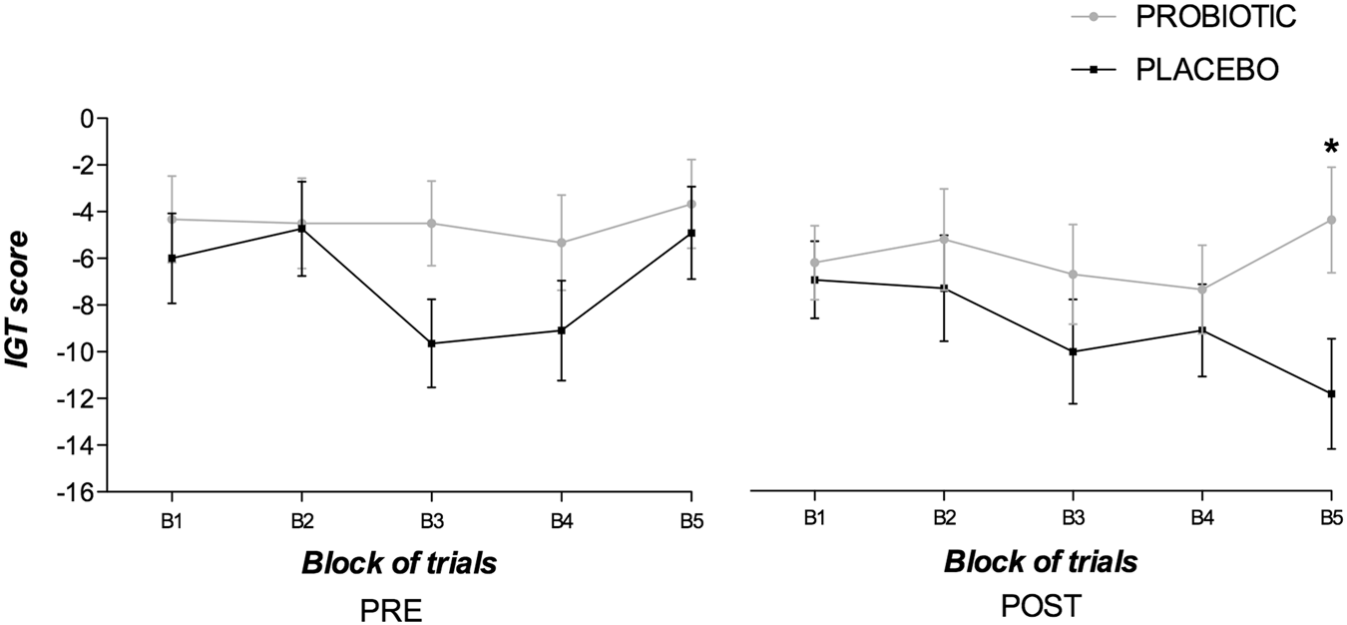
Mean net scores earned on the Iowa gambling task (IGT) by blocks of trials (total number of cards picked from the advantageous decks minus the total number of cards picked from the disadvantageous decks). Data are presented as the mean and standard error (error bars). *p < 0.05 indicates statistical significance, compared to the placebo group.

Impulsive decision-making was assessed by the IGT. The repeated measures ANOVA revealed only a marginal effect of Time [F(1, 21) = 3.82, p = 0.064, ηp^2^ = 0.154]. Given that the IGT is a learning task, although the three-way interaction did not reach significance (p < 0.05), we also conducted separate repeated-measures ANOVAs for each block. Only the analysis of the last block of 20 trials showed a main significant effect of Time [F(1, 21) = 5.55, p = 0.028, ηp^2^ = 0.209] and a marginal Group × Time interaction [F(1, 21) = 3.71, p = 0.066, ηp^2^ = 0.152]. The analysis of the interaction revealed a significantly higher number of disadvantageous cards or impulsive choices made by the control group in the post-intervention phase as compared with the probiotic group [F(1, 21) = 5.20, p = 0.033, ηp^2^ = 0.198]. This was mainly due to the control group being more impulsive after the treatment with the placebo substance [F(1, 10) = 10.52, p = 0.009, ηp^2^ = 0.513]. There were no differences between both groups in the last block of trials of the pre-intervention phase (see Fig. 2).

## Discussion

The results obtained in the present pilot study showed that both groups, probiotics and placebo, significantly decreased self-reported fibromyalgia impact (FIQ) and increased quality of life (SF-36) scores from baseline. A similar result was obtained with the affective-emotional outcome variables. That is, both groups showed a significant reduction in depressive symptoms and urinary cortisol levels at post-intervention.

Expectation of symptom improvement could be the primary factor underlying this “placebo effect”^37^. This effect has been widely demonstrated in the literature, even in people with FMS^38^. Therefore, the present results indicate that probiotic treatment did not significantly improve depressive or anxiety symptoms when compared to the placebo group. As far as we know, this is the first time that probiotics have been used with FMS patients to evaluate their impact on anxiety and mood. Previous studies have suggested the potential beneficial effects of probiotics as a preventive or adjuvant therapy for depression^13,18,39^. It is possible that in the case of people with physical and/or psychological impairments, probiotics themselves produce a beneficial effect on their emotional level (e.g., reducing anxiety and/or depression) when used as a therapeutic tool within a more comprehensive treatment framework, as suggested by Logan and Katzman^18^. This could be the case with FMS, where the symptoms of chronic, widespread and diffuse pain are often accompanied by stiffness, fatigue, sleep disorder, reduced mental performance, depression and vulnerability to the effects of negative mood^29–31,33,35–39^. The clarification of this issue may be a task for future studies.

When we considered the cognitive outcomes, response inhibition and decision-making, results showed a significant effect of probiotic treatment as compared to the placebo group. The probiotic group showed significantly fewer impulsive choices than the placebo group at post-intervention, whereas both groups had similar performance at baseline. To our knowledge, this is the first intervention study to show a reduction of impulsive behaviour in FMS patients who received a probiotic formulation for 8 weeks. This finding is in agreement with the results of a preclinical study in mice, which showed a significant reduction of obsessive-compulsive symptoms after probiotic treatment^40^.

Similar findings were obtained with the IGT, which assesses emotion-based decision-making. As shown in previous studies^35^, patients with FMS did not exhibit (either at baseline or post-intervention phase) the typical learning curve observed in healthy adults. Actually, the learning curve was flat in both the probiotic and placebo groups, which suggests a failure to learn how to optimise choices across the five blocks. However, the placebo group showed a downward tendency in performance (more disadvantageous choices at the end of the task) at the post-intervention that was not present for any of the groups at the pre-treatment assessment phase. We suggest that this downward tendency for the placebo group reflected an increase in the severity of impulsivity-related symptoms in FMS with the pass of time (two months elapsed from the pre- and post-intervention phases), which was ameliorated in the treatment group due to the effect of probiotic supplements. Future research should further investigate this hypothesis.

We also believe that the positive effect of probiotics in reducing impulsive behaviour in FMS patients may be explained in terms of the neuromodulatory effects of probiotics on serotonin and dopamine via the vagus nerve and the hypothalamus (see Collins and Bercik^41^, and Mayer *et al*.^8^, for reviews). Specifically, it has been demonstrated that probiotics can modulate the production and release of neuroactive substances such as GABA, serotonin, dopamine, acetylcholine and cytokines^42^. For instance, it has been found that some species of the genus *Lactobacillus*, even at nanomolar concentrations, trigger serotonin production in the central nervous system^42^. Similar results have been observed with some species of *Lactobacillus* in relation to dopamine production in the central nervous system^43^. Neuroanatomical evidence supports the role of the cortico-striatal-thalamic-cortical axis in impulsivity^44,45^, which has been particularly linked to alterations in serotonin and dopamine^44–47^. Future studies should be conducted to test the hypothesis that the positive effects of a probiotic treatment on impulsive behaviour might be mediated by monoaminergic alterations produced in the GBA.

The present study is not free from limitations. First, dietary measures and control for consumption of other fermented foods (e.g., yogurt) were not included. Hence, we cannot exclude that the consumption of probiotics was accompanied by spontaneous dietary changes that may have indirectly accounted for the observed effects. Second, the effect of the probiotic treatment in the microbiota was not confirmed (e.g., by stool bacterial analysis), although it should be noted that (i) previous studies have shown that the strains of probiotics included in the commercialised probiotics formula employed in the present study are able to restore the balance of the gut flora; and (ii) studies using partly the same bacterial strains as those used in the present study have confirmed the presence of such strains in stool samples of healthy volunteers^48^. Therefore, we propose that further studies should control participant’s diet habits during treatment and measure the effect of probiotics on microbiota in a larger sample of patients to replicate and extend the present findings of a positive effect of probiotics on impulsive behaviour. Future studies might also manage the high placebo effect that we observed in some of the emotional measures by the recruitment of greater number of patients or more severely affected patients^49^.

To conclude, the present findings demonstrate, for the first time, that an 8-week multispecies probiotic intervention improves cognition, specifically impulsive choice and decision-making, in a group of patients diagnosed with FMS. No others beneficial effects were observed in self-reported pain, quality of life, fibromyalgia impact, depressive or anxiety symptoms. Future studies should investigate the effects of probiotics in combination with other treatments, including dietary changes, in FMS and other clinical populations, as well as the impact of these live organisms on other cognitive functions affected in FMS patients (e.g., cognitive flexibility or working memory).

## Materials and Methods

### Study Design

This was a double-blind (group allocator, participants, outcome assessor) randomised controlled intervention pilot study, with a placebo control group and an experimental group. The intervention study lasted for 8 weeks, from December 2015 to February 2016.

This study was part of a larger study^50^ that received ethics approval by the Human Research Ethics Committee of the University of Almeria (Spain). The intervention study was registered with ClinicalTrial.gov (NCT02642289; December 30, 2015). Here, we report the results from two cognitive tasks to evaluate impulsive choice and decision-making as well as those from mood and functional state self-report questionnaires in two groups of participants (Probiotic 2 and control). Informed written consent, including information about the different types of intervention (probiotic vs. placebo), was obtained from all participants. This study was conducted in accordance with the Declaration of Helsinki and the Consolidated Standards of Reporting Trials (CONSORT) see electronic supplementary information.

### Participants

**Figure 3:**
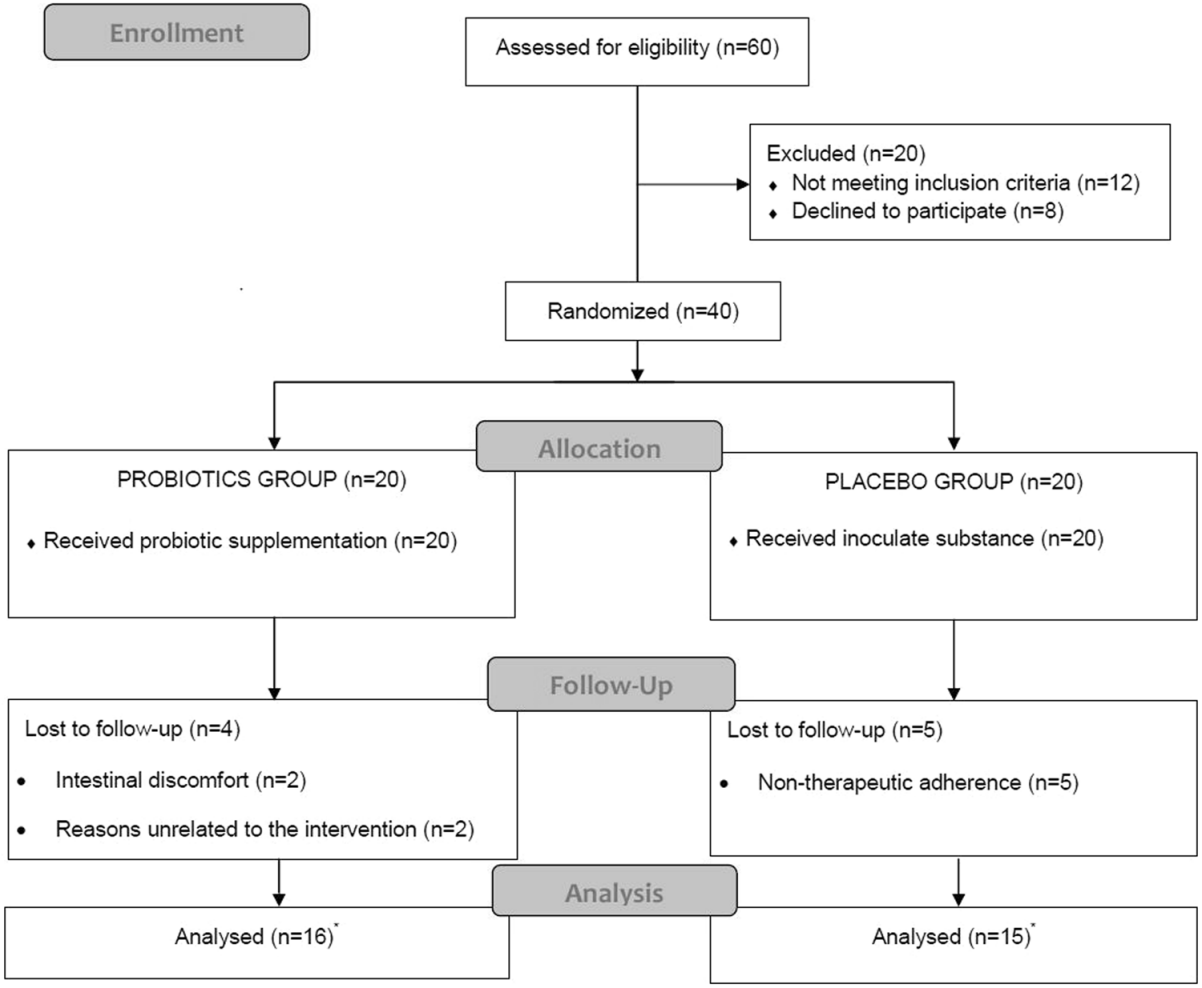
Summary of patient flow diagram according to Consort’s requirement. *See “Statistical analyses” for more details about analysed participants in each group.

Forty patients diagnosed with FMS according to the criteria of the American College of Rheumatology (ARC)^19,20^ participated in the study. Fibromyalgia patients were recruited from the Almeria Fibromyalgia Association (AFIAL-Spain) or from El Ejido Fibromyalgia Association (AFIEL-Spain) and had been diagnosed at least 1 year before entering the study by a relevant clinician who in accordance to Wolfe *et al*.^21^ employed both ACR criteria, from 1990 and 2010. Exclusion criteria included: (1) using antibiotics and nutritional supplements, (2) allergies, (3) currently participating in other psychological or medical studies, (4) being pregnant or breastfeeding, (5) severe intestinal disease, and (6) meeting the criteria for psychiatric disorders other than depression and/or anxiety. An initial telephone or personal interview was conducted to ensure that the participants fulfilled the basic study criteria. A total of 60 people were assessed for eligibility; 8 refused to participate, and 12 were excluded because they did not meet the inclusion criteria (see Fig. 3).

Each participant was randomly assigned to either the placebo or the intervention (probiotic) group through a random number generator. Twenty participants (1 male) with a mean age of 55.00 ± 8.37 years and a mean body mass index (BMI) of 29.4 ± 6.57 kg/m^2^ were assigned to the probiotic condition, and 20 participants (2 males) with a mean age of 50.27 ± 7.86 years and a mean BMI of 30.23 ± 6.31 kg/m^2^ were assigned to the placebo condition. During the 8 weeks, four participants in the probiotic group abandoned the study (two of them due to intestinal discomfort, and the other two for reasons unrelated to the intervention), and five participants in the placebo group abandoned the study due to non-adherence to treatment. Thus, the final sample included 16 participants in the probiotic condition and 15 participants in the placebo condition (see Fig. 3).

### Procedure

In the probiotic intervention group, participants were given four bottles, without identification, of ERGYPHILUS Plus (Laboratorios NUTERGIA S.L., Spain) containing 60 pills each (6 million revivification of germs per capsule). ERGYPHILUS Plus contains the following bacterial strains: *Lactobacillus Rhamnosus GG*^*®*^*, Casei, Acidophilus*, and *Bifidobacterium Bifidus*. In the placebo intervention group, participants were also given the same four bottles containing 60 pills each; the pills (composed of cellulose) were indistinguishable from the probiotics in colour, taste and smell (provided by Complementos Fitonutricionales –CFN- S.L., Spain).

All participants were evaluated pre- (baseline) and post-intervention, for pain, impact of fibromyalgia, anxiety, depressive symptoms and quality of life. Their performance in two impulsivity tasks was also assessed. Finally, pre- and post-treatment urine free cortisol levels were measured using a first urine sample collected in a sterile container and conserved at −20 °C. Each participant was instructed to refrain from ingesting medication which they could stop without significant health consequences and that could affect cognitive performance (for example, opioid analgesics) at least 48 h before the assessment, and from 3 hours prior to the assessment any CNS stimulant or depressant (for example, alcohol, tobacco and coffee).

A week after the pre-intervention assessment, participants were given two bottles (containing either the inert placebo or the multispecies probiotic) for the first 4-week intervention. Three weeks later, they were given the other two bottles for the rest of the intervention. Participants (both placebo and probiotic) received detailed dosing schedule instructions. They were required to take two pills at least 30 minutes before breakfast and two pills at least 30 minutes before dinner. They were also instructed to store the bottles in the refrigerator.

### Outcome measures

#### Demographic and questionnaires

All participants provided the following demographic and clinical information: gender, age, FMS diagnosis onset, years of formal education and body mass index (BMI) that was calculated by dividing the weight (in kilograms) by the square of height (in meters).

Pain was assessed with the Visual Analogue Scale (VAS)^51^, a widely used unidimensional measure of pain intensity. The score ranges from 0 (no pain) to 10 (worst imaginable pain). The VAS has shown an internal consistency of 0.56 to 0.88^52^.

The impact of fibromyalgia was measured by the Fibromyalgia Impact Questionnaire (FIQ; Burckhardt *et al*.^53^; Spanish version by Monterde *et al*.^54^), a self-administered questionnaire that consists of 21 individual questions that assess perceived pain intensity in the context of the past 7 days. Each question is answered using a Likert-like scale from 0 to 10. The FIQ total score ranges from 0 to 100, with higher scores indicating greater impact of the condition on the person’s life^55^.

The SF-36 Quality of Life Questionnaire (Ware and Sherbourne^56^; Spanish version by Alonso *et al*.^57^) was employed to measure participants’ quality of life. The questionnaire consists of 36 items grouped into eight domains: physical, social, emotional, mental health, vitality, overall health, and changes in health over time. Scores for each sub-domain range from 0 (worst health status) to 100 (optimal health status). Studies have reported good internal consistency for the SF-36, the α coefficient ranges from 0.78 to 0.96^57^.

Anxiety levels were measured with the 40-item State-Trait Anxiety Inventory (STAI; Spielberger *et al*.^58^; Spanish version by Seisdedos^59^), which measures state and trait anxiety separately. Participants are asked to report the intensity and frequency of their feeling of anxiety using a Likert-like scale from 0 (*not at all* for intensity, and *almost never* for frequency) to 4 (*very much so* for intensity and *almost always* for frequency). Studies have reported good internal consistency for the two scales, α coefficient from 0.90 to 0.93 for the STAI-state and from 0.84 to 0.91 for the STAI- trait^58^.

The presence and severity of depressive symptoms were measured with the Beck Depression Inventory (BDI; Beck *et al*.^60^; Spanish version by Conde and Useros^61^). This is a self-reported questionnaire that consists of 21 items that assess the intensity of depressive symptoms using a Likert-like scale from 0 (symptom not present) to 3 (symptom very intensive)^62^. The total score ranges from 0 to 63, with higher scores indicating higher levels of depression. The test-retest reliability of the BDI is good (r ranges from 0.65 to 0.72) and so is the internal consistency (α = 0.82)^63^.

Participants also completed the adapted and validated Spanish version of the Mini-Mental State Examination (MMSE)^64^, a screening measure of global cognitive function. The score ranges from 0 to 30, with a cut-off score of 24 for potential dementia diagnosis.

#### Cognitive tasks

Participants performed two computerised cognitive tasks widely used to assess impulsive choice and decision-making; the two-choice task and the Iowa gambling task, respectively. E-Prime 2.0 software (Psychology Software Tools, Pittsburgh, PA) controlled the presentation of the stimuli as well as collection of the participant’s responses in the tasks. Stimuli were presented on a dark background on a colour monitor (VGA) of an IBM/PC compatible computer.

The two-choice task measures the tendency to choose a small reward over a larger delayed reward^65^. In this study, participants were presented with 40 trials in which they could choose to wait 5 s for five points or 15 s for fifteen points. On each trial, a diamond and a star appeared on the screen. Participants were required to select one of the two shapes. The length of the delay and the size of the reward associated with each shape remained constant across the testing session; for example, the star allowed the participant to earn 5 points after waiting 5 s, while the diamond was followed by 15 points after waiting for 15 s. Impulsive choices were defined as the total number of short-delay reward responses.

A computerised version of the original Iowa gambling task (IGT; Bechara *et al*.^66^) was used as a measure of impulsive decision-making. This task measures risk sensitivity, as defined by the inability to anticipate and reflect on the consequences of decision-making. Participants made a total of 100 card selections from four decks of cards labelled A, B, C and D. They were instructed to select one card, with the goal of obtaining the highest amount of virtual money. Decks A and B produced higher returns than C and D, but also greater losses, so the net result is earning less virtual money. Thus, options A and B are disadvantageous, while C and D are advantageous. The analysis is conducted by grouping the 100 trials of the task into blocks of 20 trials each and calculating an IGT-index for each block by the following formula: (C + D) − (A + B).

#### Physiological measures

Urine samples were tested for their concentration of cortisol using a competitive ELISA kit (RE5224, Cortisol ELISA, IBL International, Hamburg, Germany) with a DU 530 Beckman spectrophotometer. The ELISA kit is a competitive immunoenzymatic colorimetric method for quantitative determination of free cortisol concentration in urine. The lowest detectable concentration of urinary cortisol that can be distinguished from the zero standard is 2.0 ng/mL at the 95% confidence limit, according to the manufacturer. The assay was performed according to the instructions and the procedure provided by the manufacturer.

### Statistical analyses

Statistical analyses and graphics were performed using SPSS v19.0 (SPSS, Inc., Chicago, IL) and GraphPad Prism v7.0 (GraphPad Software, La Jolla California USA), respectively. All alpha levels were set at p < 0.05. As this was a pilot study, no power analysis was performed to pre-determine sample size.

First, a descriptive analysis was performed and the normal distribution of variables was verified by the Kolgomorov-Smirnov test. Baseline demographics were compared between both groups using χ^2^ tests for categorical data and Student t-tests for continuous data. For questionnaires and behavioural measurement of impulsivity, the mean scores (total and/or partial) were submitted to a repeated measures analysis of variance (ANOVA) with Time (pre- vs. post-intervention) as the within-subjects factor and Group (probiotic vs. placebo) as the between-subjects factor. In addition to the statistical significance levels, the effect size estimates were calculated by ηp^2^ (*partial eta-squared*). This indicator provided estimates of the magnitude of the effects that are independent of the sample size^67,68^.

Due to technical problems, four participants assigned to the probiotic group and four participants assigned to the placebo group were missing in the IGT as well as five participants from the placebo group and three from the probiotic group in the two-choice task. Data from one participant assigned to the probiotic group were also deleted from the cortisol measure. No other data were missing.

All data analysed during the current study are available from the corresponding author on reasonable request.

## Electronic supplementary material

Supplementary Information
